# Bilateral Conjunctival Infiltration as an Extramedullary Relapse of AML

**DOI:** 10.1155/2018/9590469

**Published:** 2018-02-12

**Authors:** Zahra Mozaheb, Alireza Khooei

**Affiliations:** Mashhad University of Medical Sciences, Mashhad, Iran

## Abstract

Acute myelogenous leukemia (AML) accounts for 1.2% of all cancer deaths. Relapse is the major cause of treatment failure in acute myeloid leukemia (AML) patients. AML rarely presents as ocular manifestation in relapse or at presentation. The M4 subtype of AML is most commonly presented with extramedullary involvement. In this report, we presented a young female with AML who was diagnosed and treated for AML about 40 months ago. She did not transplant because she did not have a full-match donor. About 4 months ago, she visited with a red eye and conjunctival infiltration. She was referred to an ophthalmologist for a biopsy, and the pathology report showed the relapse of AML which was treated with systemic chemotherapy. Red eyes with subconjunctival nodules in patients with a history of previous AML should raise the suspicion for recurrent disease that warrants urgent biopsy and systemic treatment. Eye involvement with leukemia is usually responsive to systemic chemotherapy.

## 1. Introduction

Long-term survival rates in acute myeloid leukemia have been markedly improved by intensification of first-line treatment and better supportive care with overall survival rates increasing from 40 to 60% during the past two decades. However, relapse remains a major obstacle for improving prognosis further, and also, we can see unusual relapse. AML has only rarely been reported as causing ocular complications. Most ocular infiltrates from AML include chorioretinal or optic nerve lesions and are found incidentally in patients with advanced disease [1, 2]. Based on our searching, this is the first report of AML relapse with bilateral conjunctival infiltration, pathologically showing leukemic infiltration.

## 2. Case Presentation

An 18-year-old woman with a history of documented AML was admitted in Imam-Reza Hospital in Mashhad (Iran) in December 2014. The bone marrow specimen consisted of more than 70% myelomonoblast (M4), which was positive for MPO, CD13, CD14, and CD33 in flow cytometry study, and also she had normal cytogenetics. She underwent induction chemotherapy with 3 + 7 regimen daunorubicin + ara C and achieved complete remission, after that she took intensification chemotherapy for 3 cycles. She was candidated for hematopoietic stem cell transplantation, but she was a single child and did not have a full-match donor. Therefore, we searched for unrelated full-match donor, but we could not find one and she underwent 6-thioguanine as maintenance.

**Figure 1:**
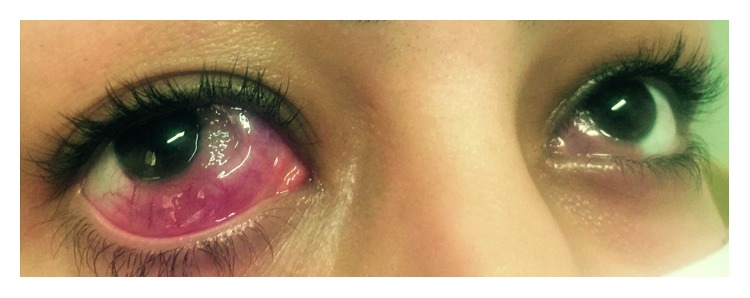
Bilateral conjunctival infiltration as the first sign of AML relapses.

She presented with complaints of painful and bilateral red eyes after three and half years (Figure 1). She had been in her usual state of health until approximately 3 weeks prior to the admission, when she began to develop red eyes, photophobia, and pain in the eyes. In the course of systemic evaluation, she did not have any other sign or symptoms that showed the systemic relapse. Ophthalmic examination revealed normal visual acuity and extraocular movement, but marked scleral and conjunctival lesion of bilateral eyes was noted. Other workups including cerebrospinal fluid and cranioorbital magnetic resonance imaging study were normal.

**Figure 2:**
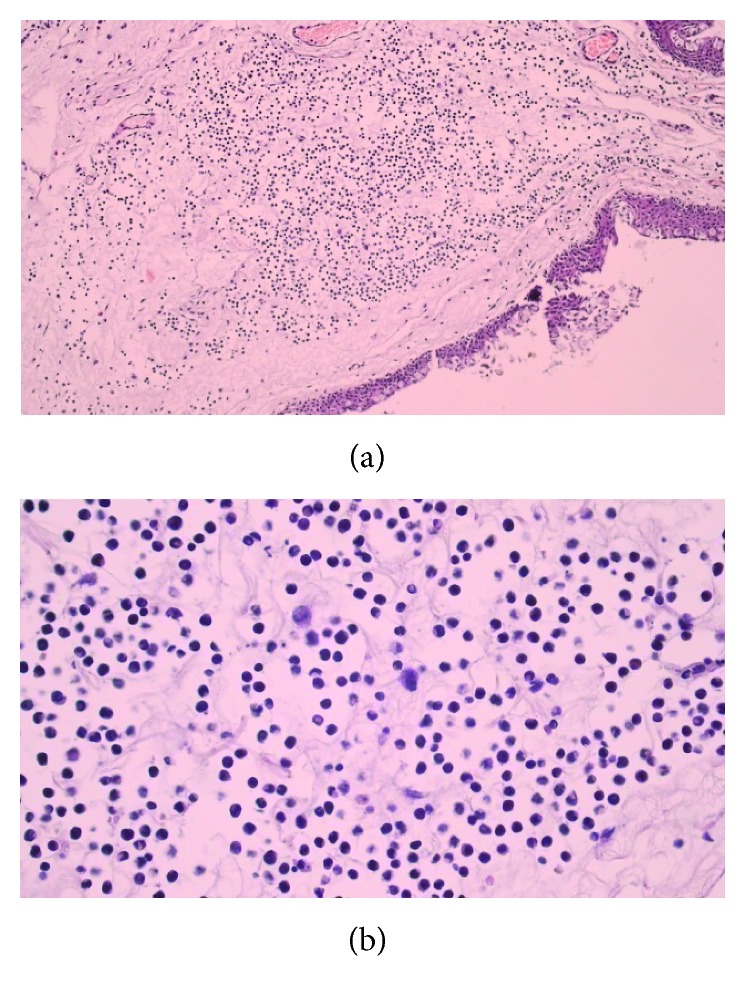
(a) Conjunctival infiltrate of neoplastic cells, H&E, 40x. (b) Conjunctival infiltrate of neoplastic cells which show hyperchromatic nuclei, some with nuclear indentation. Few blasts are also observed, H&E, 400x.

**Figure 3:**
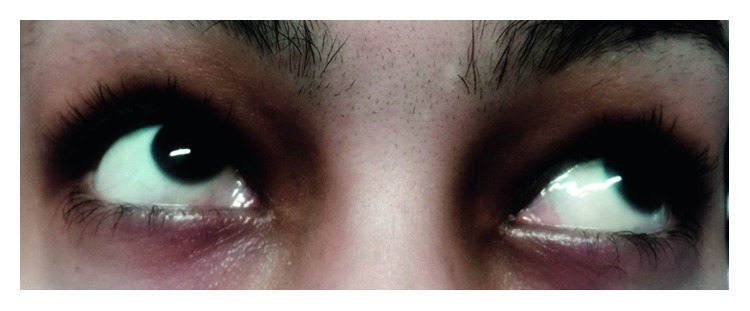
The patient's eyes after induction chemotherapy.

Her leukocyte count and differentiation, Hb, and platelet count were normal. Peripheral blood smear and bone marrow examination were done, and she was in complete remission. Biopsy was taken from her conjunctival lesion, and infiltration of blast cells was reported (Figure 2). Subsequent examination of the sample by flow cytometry was consistent myeloblast with MPO and CD117 positivity. She underwent chemotherapy with 3 + 7 regimen daunorubicin + ara C, and we can see her eyes after induction chemotherapy in Figure 3. After that, she was taking 4 cycles of high-dose ara C as consolidation.

## 3. Discussion

In patients with acute leukemia, it has been necessary to take a closer look at the site of extramedullary leukemic infiltration because of local morbidity and also because they might act as a reservoir for proliferation of leukemic cells and eventual systemic relapse. Ophthalmic findings are rare findings as an initial manifestation of new or relapsed disease [3]. Eyes are the only site where the leukemic involvement of nerves and blood vessels can be directly observed; therefore, knowledge of ocular involvement in leukemia is important [4].

The M4 myeloid leukemia subtype consists of about 20% of all AML cases, and it is associated with extramedullary infiltration most frequently in gingiva, skin, and central nervous system in comparison to other subtypes [5]. Ocular manifestation was reported about 3% in ALL patients [6], but AML has only rarely been reported as finding ocular complication at presentation or relapse [1]. The survival of patients with acute leukemia has considerably improved with evolving diagnostics and therapeutics. This has led to an increase in the variability of unusual site of relapse such as ocular presentation as a first sign of relapse [7].

In two prospective studies of 116 patients diagnosed with AML, none of them were found to have leukemic infiltration in their eyes. Ocular manifestations of leukemia may present as direct infiltration of the uvea, conjunctiva, and optic nerve or as secondary to the hematologic changes of leukemia such as thrombocytopenia, anemia, opportunistic infections, and hyperviscosity [8]. Chorioretinal or optic nerve lesions are the most ocular infiltration in AML, and they are found in the advanced disease [2, 9]. Leukemic involvement of the eye in different studies has varying prevalence, ranging from 50 to 80% and a higher incidence of involvement in histopathologic postmortem studies [10, 11]. Central nervous system is one of the most frequent sites of relapse, after initial induction of remission [12], and also recent studies showed that there is a close correlation between eye involvement in acute leukemia and central nervous system disease [13].

## 4. Conclusion

Red eyes with subconjunctival nodules in patients with a history of previous AML should raise the suspicion for recurrent disease that warrants urgent biopsy and systemic treatment. It is suggested that eye relapse occurred because it may be an immune-privileged site such as the eyes, testis, and brain for AML relapse and will further highlight the importance of this site for cancer relapse. If there is a high suspicion for relapsed leukemia, systemic treatment is often integral to resolution of the ocular problems. It is usually responsive to systemic chemotherapy, local radiation treatment to the eye, or a combination of both of them.
